# Supplementary material for: Establishment and Characterization of hTERT Immortalized Hutchinson–Gilford Progeria Fibroblast Cell Lines
Source: Cells. 2022 Sep 6;11(18):2784. doi: 10.3390/cells11182784 (PMC9497314; doi:10.3390/cells11182784)
Supplement: Supplementary file 1 [file cells-11-02784-s001.zip › cells-1839461-supplementary.pdf]

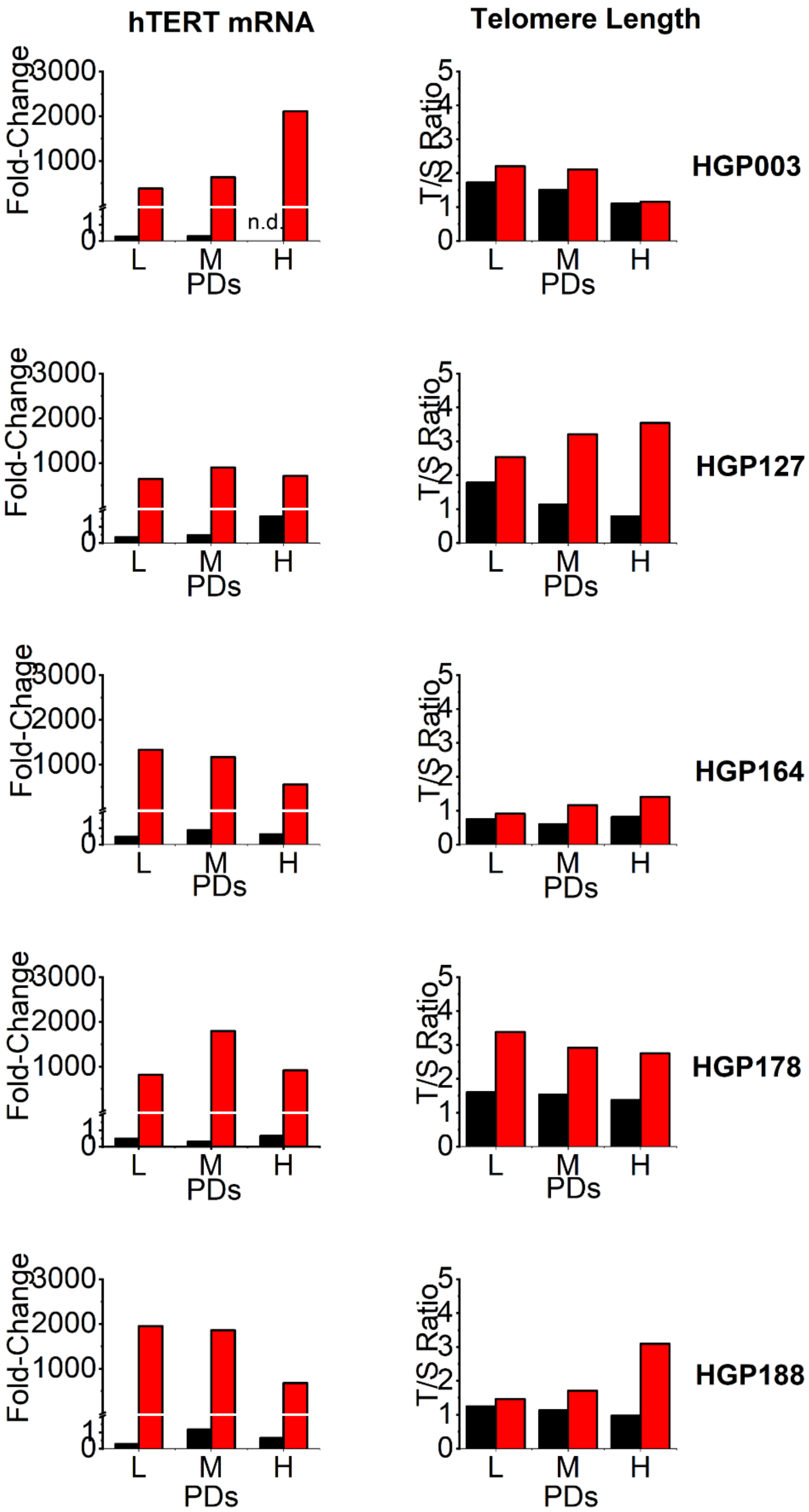


**Figure S1. *hTERT* mRNA expression, and telomere length of HGP fibroblasts.** mRNA expression: given as mRNA values relative to a standard (N17 at low PD), as described in Materials and Methods. Telomere length: T/S ratio represents the ratio of the content of telomere (T) to a single-copy gene (S). L, low PD; M, medium PD. H, high PD. The values represent the means of technical triplicates.

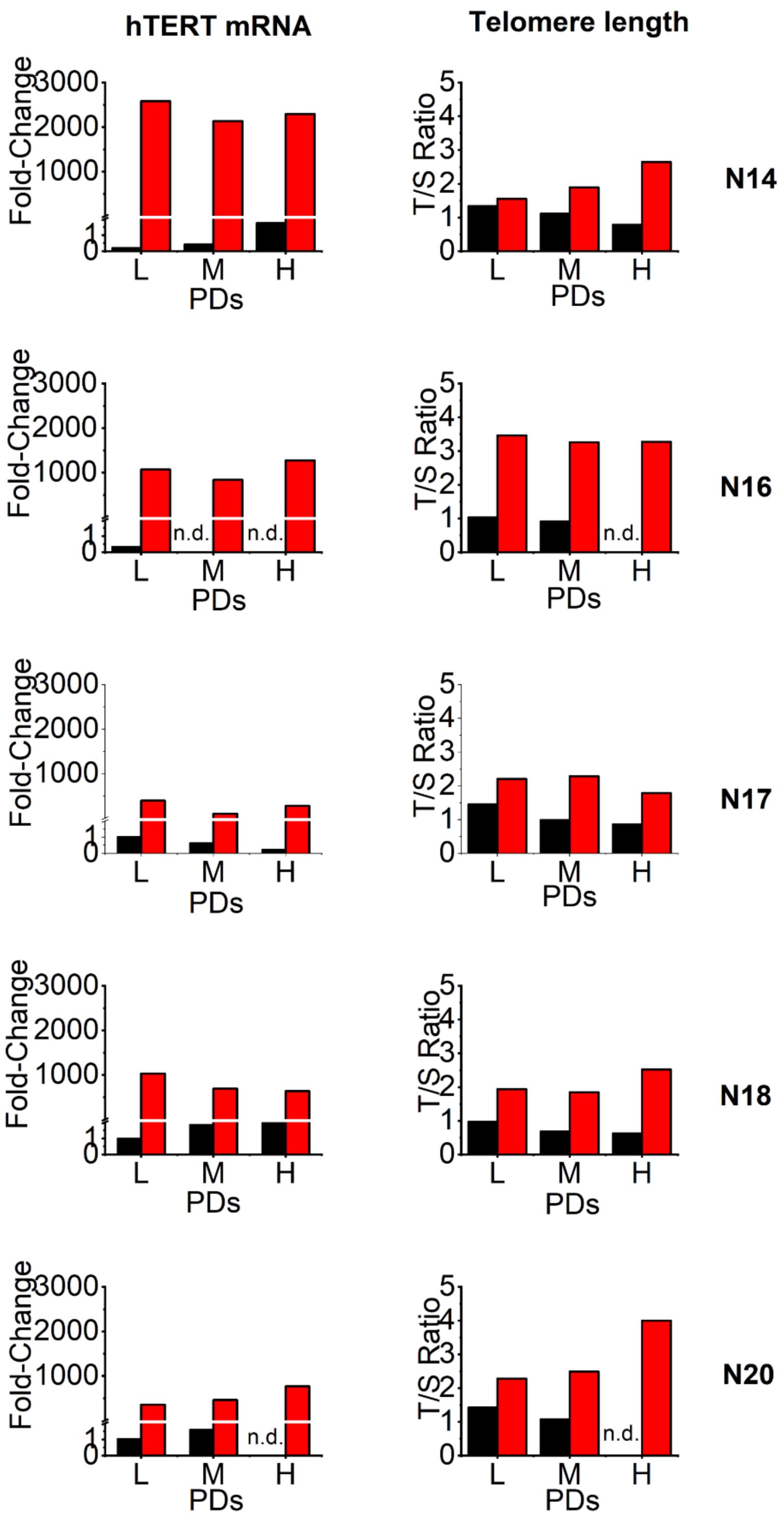


**Figure S2. hTERT mRNA expression, and telomere length of healthy control fibroblasts.** mRNA expression: given as mRNA values relative to a standard (N17 at low PD), as described in Materials and Methods. Telomere length: T/S ratio represents the ratio of the content of telomere (T) to a single-copy gene (S). L, low PD; M, medium PD. H, high PD. The values represent the means of technical triplicates. N.d., not detectable due to insufficient cell material of pre-senescent cells.

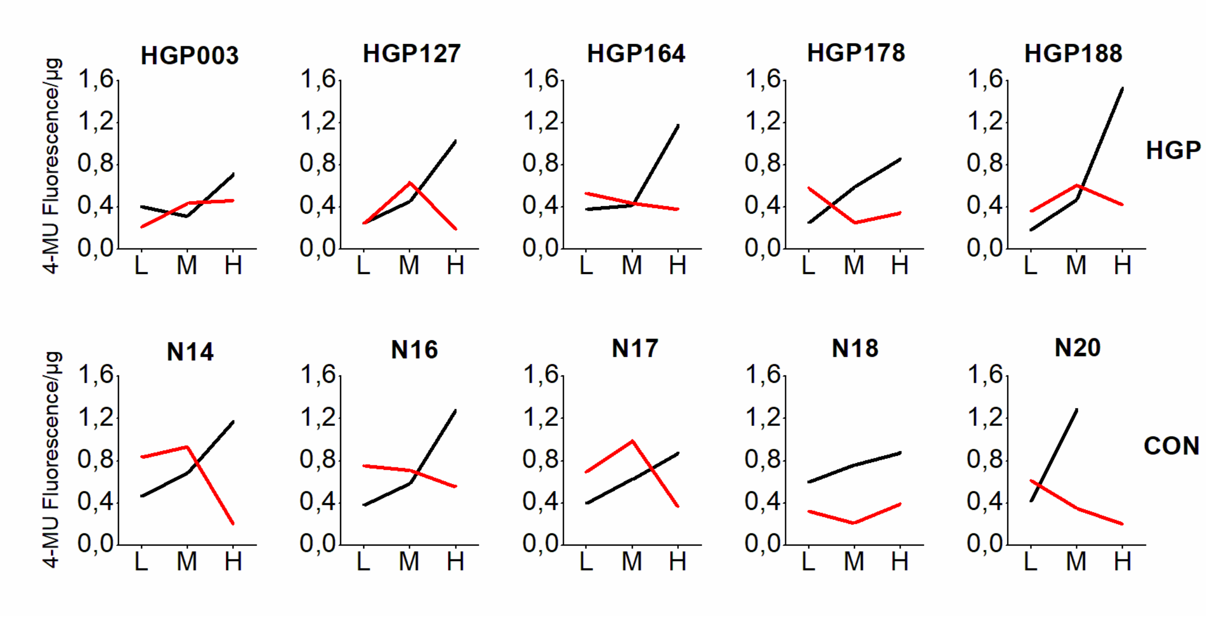


**Figure S3.** Quantification of SA-β-gal activity (4-MU fluorescence) of HGP and control fibroblasts before (black line) and after (red line) immortalization. Cell extracts were prepared as described in the Method section. Extracts were incubated with MUG fluorogenic substrate at 37 °C, and aliquots were removed from the reaction mixture at three hours to measure the fluorescence intensity of the hydrolysis product 4-MU. Readings were normalized for total protein per assay to correct for differences in extract concentration. Assays were run in duplicates with means plotted. L, low PD; M, medium PD. H, high PD.
